# Intra-Rater Reliability of Rehabilitative Ultrasound Imaging for Multifidus Muscles Thickness and Cross Section Area in Healthy Subjects

**DOI:** 10.5539/gjhs.v7n6p354

**Published:** 2015-07-07

**Authors:** Mohammad Hosseinifar, Asghar Akbari, Fateme Ghiasi

**Affiliations:** 1 Health Promotion Research Center, Department of Physiotherapy, School of Rehabilitation Sciences, Zahedan University of Medical Sciences, Zahedan, Iran

**Keywords:** rehabilitative ultrasound imaging, reliability, multifidus muscle, pelvic floor muscle

## Abstract

**Introduction:**

Rehabilitative Ultrasound Imaging (RUSI) must be valuable method for research and rehabilitation. So, the reliability of its measurements must be determined. The purpose of this study was to evaluate the intra-rater reliability of RUSI for measurement of multifidus (MF) muscles cross section areas (CSAs), bladder wall diameter, and thickness of MF muscles between 2 sessions in healthy subjects.

**Method:**

Fifteen healthy subjects through simple non-probability sampling participated in this single-group repeated-measures reliability study. MF muscles thickness at rest and during contraction, MF muscles CSAs at rest, and bladder diameters at rest and during pelvic floor muscles (PFM) contraction were measured through RUSI. Pearson’s correlation coefficient test was used to determine intra-rater reliability of variables.

**Finding:**

The results showed that intra-class correlation Coefficient (ICCs) values with 95% confidence interval (CI) and the standard error of the measurement (SEM) were good to excellent agreement for a single investigator between measurement occasions. The intra-rater reliability for the bladder wall displacement was high (ICCs for rest and PFM contraction state: 0.96 and 0.95 respectively), for the MF muscles CSAs at the L4 level was good to high (ICCs 0.75 and 0.91 for right (Rt) and left (Lt) side respectively), and for the thickness of MF muscles at two levels, at rest and during two tasks was moderate to high (ICCs: 0.64 to 0.87).

**Conclusion:**

The Trans-Abdominal (TA) method of RUSI is a reliable method to quantify the PFM contraction in healthy subjects. Also, the RUSI is a reliable method to measure the MF muscles CSAs, the MF muscles thickness at rest and during functional tasks in healthy subjects.

## 1. Inroduction

Lack of core stability is supposed as one of the important predisposing cause of recurrent low back pain (LBP) (George et al., 2007). Increasing attention have been paid to train preferentially localized spinal stabilizer muscles in subjects with LBP (Danneels et al., 2001). The altered function of the trunk deep stabilizing muscles affects on spinal stability. So, the subjects with lumbo-pelvic pain have muscular dysfunction and motor control deficit of deep local muscles (Richardson et al., 2004). The lumbar multifidus (MF) muscles have been proposed to play an important role in spinal stability (Hodges PW and Richardson CA 1999; Hungerford B, Gilleard W et al. 2003). It have been shown that there is functional deficits of MF muscles in individuals with LBP (Hides et al., 1994; Hodges et al., 2006). Also, PFM are accepted as a part of the trunk stability mechanism (Hodges et al., 2005). Pelvic floor muscle (PFM) dysfunction has been related to the development of lumbo-pelvic pain (Whittaker, 2004). Since, PFM and MF muscles contributed for trunk stability through feed-forward activation in response to trunk perturbation, the role of these muscles is important to stability of the spinal column (Hodges et al., 2005).

Rehabilitative ultrasound imaging (RUSI) has been advocated as a noninvasive method to quantify muscle morphology and function (Rasouli Omid et al., 2011). This method permits assessment of muscle activity by measuring changes in muscle geometry during contraction (Kiesel et al., 2008). RUSI has been validated as a tool to determine muscle morphology through comparisons with magnetic resonance imaging measurements (Hides et al., 1995) and as an indicator of muscle activation with needle electromyography (Kiesel et al., 2007).

Since RUSI must be valuable method for research and rehabilitation, the reliability of its measurements must be determined. Although, the application of RUSI for imaging the pelvic organs and structures is not new method, a few studies conducted to evaluate PFM function (Sherburn et al., 2005; Thompson et al., 2005).

Sherburn et al. (2005) investigated the intra-rater reliability of TA approach of RUSI of PFM contractions through posterior bladder wall displacement on non-pregnant adult female subjects aged 24 to 57 years. Average intra-class correlation (ICC) coefficients for intra-rater reliability was between 0.81 and 0.89 (Sherburn et al., 2005). Also, Thompson et al. (2005) assessed the reliability of TA and trans-perineal (TP) ultrasound during a pelvic floor muscle (PFM) contraction on 10 women. The reliability during PFM was excellent for both methods (Thompson et al., 2005).

Several researchers have investigated the reliability of RUSI to measure the thickness of the lumbar MF muscles (Hides, 1992; Hides et al., 1995; Hodges et al., 2006; Van Khai et al., 2006; Kiesel et al., 2007; Kiesel et al., 2008; Wallwork et al., 2007; Koppenhaver et al., 2009). However, we can’t find any researches that investigated the reliability of RUSI to measure MF muscles thickness at two vertebral levels during two functional tasks with involuntary contraction of the MF muscles. Koppenhaver et al. (2009) evaluated the intra-examiner and inter-examiner reliability of the RUSI in obtaining thickness measurements of the lumbar MF muscles at rest and during contractions in patients with LBP at L4/L5 level. They concluded that there are high intra-examiner reliability and good inter-examiner reliability for thickness measurements of the lumbar MF muscles (Koppenhaver et al., 2009). Wallwork et al. (2007) investigated intra-rater and inter-rater reliability (1 experienced and 1 novice tester) of lumbar MF muscles thickness in para-sagittal section in healthy subjects at rest by using real-time ultrasound imaging. They concluded that when a standardized protocol was used, there were high inter-rater and intra-rater reliability (Wallwork et al., 2007).

The intra- rater reliability of RUSI for measurement of lumbar MF muscles cross section areas (CSAs) were investigated by Stokes et al. (2005) at L4 level of lumbar spine on ten normal subjects at the left side (Stokes et al., 2005) and by Pressler et al. (2006) at the bilateral side of S1 level within 1 to 4 days for repeated measurements (Pressler et al., 2006). They found moderate to high intra-rater reliability for RUSI for measurement of MF CSAs of the lumbar MF muscles.

So, the purpose of this study was to evaluate the intra-rater reliability of RUSI for measurement of MF muscles CSAs at two sides of L4 level of lumbar spine, bladder wall displacement as a reflection of PFM action, and thickness of MF muscles at rest and during contractions at two level of lumbar vertebrae between 2 sessions (between two day) in healthy subjects. Therefore, in the present study several outcome measures; MF muscles thickness, MF muscles CSAs, and PFMs activation were investigated simultaneously in healthy subjects. Our study design and testing procedures were different from previous studies aspects of participants include from both gender and without LBP, MF muscles thickness were evaluated at two levels of lumbar spine and during two functional tasks and all outcome measure of MF muscles were measured at two side of lumbar spine.

## 2. Methods

### 2.1 Study Design

This study was a Single-group repeated-measures reliability study. Fifteen healthy subjects through simple non-probability sampling participated in this study. This study was done at Physiotherapy Clinic, Zahedan University of Medical Sciences, between June and August 2014. All participants signed written informed consents.

### 2.2 Participants

Fifteen healthy subjects with age between 22-42 years participated in this study. Their demographic characteristics are shown in Table 1. Inclusion criteria were: without history of LBP requiring medical care or time off work in the past 10 years, subjects were excluded if they had current or previous LBP, a history of trauma or previous surgery at lumbar region, spondylolysis or spondylolysthesis, spina bifida, spinal stenosis, pregnancy, cardiovascular diseases (Kiesel KB, Uhl TL et al. 2007; Mannion AF, Pulkovski N et al. 2008).

**Table 1:** Demographic characteristics of subjects

	Mean and Standard Deviation	Range
**Age (y)**	27.06±6.80^b^	22-42
**Height (cm)**	170.93±8.60	161-195
**Weight (kg)**	65.69±7.23	55.6-75.5
**BMI^a^**	22.50±2.16	19.46-24.84

a BMI= body mass index,

b Values are Means and Standard Deviation

### 2.3 Data Collection

Firstly, demographic data; Age, height, weight, and body mass index (BMI) were measured. MF muscles thickness at rest and during contraction, MF CSAs at rest, and bladder diameters at rest and during PFM contraction were measured through RUSI based on the following procedures:

### 2.4 Ultrasound Recordings

To measure the thickness and CSAs of MF muscles, and bladder diameters, the B-mode ultrasound apparatus (ESAOTE S.p.A, My Lab X Vision 50, Italy) was used. All ultrasound recordings were done at two times, with 2 days interval by one operator. At each time, three repetitions of each of outcome measure were done and mean value of three measurements was taken for statistical analysis. MF muscles thickness was measured at two level; L3-4 and L5-S1 on both sides. To randomized ultrasound assessment, for each of subjects, the side and level of measurement randomly selected. Also, the sequences of recording of MF muscles thickness, CSAs, and bladder diameter randomly selected.

#### 2.4.1 Ultrasound Recordings of the MF muscles Thickness

Thickness measurement of MF muscles was performed in resting position and during two tasks with sub-maximal muscle contraction in both sides (Teyhen et al. 2005). To record the thickness of MF muscles, we used a CA 431 curvilinear probe set to 7.5 MHz. Measurements of the MF muscles thickness were performed in the prone position with a pillow under the abdomen. The probe was placed along the spine, in a manner that the mid-point of the probe was in line of spinous process of the lumbar vertebra. Then, it was moved to visible the facet joint between the two lumbar vertebrae. This point is located directly on MF muscles. The thickness of the MF muscles was measured at the levels of L3-4 and L5-S1 zygapophyseal joints using on-screen calipers. Two vertebral levels were selected, because the morphology of the MF muscles varies according to vertebral level. Linear measurements were conducted from the tip of the target zygapophyseal joint to the inside edge of the superior border of the MF muscles (Kiesel et al., 2008). The sub-maximal tasks for this muscle were elevation of the contra-lateral arm in prone position without weight (task1) and with small weight (0.5 kg) on hand (task2), elbow in right angle, and gleno-humeral joint in 120 degrees of abduction (Hebert et al., 2010).

#### 2.4.2 Ultrasound Recordings of the MF muscles CSAs

MF muscles cross section areas were measured at L4 vertebral level. During measurement, subjects try to relax the para-vertebral musculature. After relaxation, the probe placed transversely over the L4 spinous process. With this probe placement L4 spinous process and lamina must be seen. So, MF muscles could be seen on both sides of the spine. Vertebral lamina was used as a landmark to identify deep border of the MF muscles. To assess the CSAs of the MF muscles, we need to bordered MF muscles superiorly by the thoracolumbar fascia, medially through the spinous process of L4 vertebrae, and laterally by the fascia between MF and longissimus group muscles (Wallwork et al., 2009).

#### 2.4.3 Ultrasound Recordings of the Bladder diameters (TA Approach)

Bladder diameters at rest and during PFM contraction was measured through the CA 431 curvilinear probe set to 3.5 MHz. The subjects were tested in a crook-lying supine position with one pillow underneath the head (Chehrehrazi et al., 2009). The hips and knees were flexed to 60°, and the lumbar spine was positioned in neutral (Chehrehrazi et al., 2009).To visualize the bladder base, a standardized bladder filling protocol was used prior to imaging; drinking 600–750 ml of water during half an hour prior to testing, without voiding during this period (Sherburn et al., 2005). Amounts of the bladder base movement on ultrasound image was used as indicator of PFM contraction based on the TA method (White et al., 1980). Ultrasound probe was placed in the transverse plane immediately superior of the pubic bone and angled in a caudal/posterior direction to obtain an optimal image of the inferior–posterior aspect of the bladder. Subjects were required to perform PFM contraction through instruction to draw in and lift the PFM and to keep the contraction while breathing normally. Then, the image was fixed, and the subjects relaxed. The amounts of the bladder diameter at rest and at the end of each contraction was measured in millimeter (mm) (Sherburn et al., 2005; Thompson et al., 2007; Chehrehrazi et al., 2009). Subjects performed three maximal contractions without movement of the pelvis or lumbar spine and without palpable contraction of muscles of pelvic or abdominal region. All contractions were held for 3 second with a rest of 10 second between each contraction.

### 2.5 Statistical Analysis

Results were presented as mean values and standard deviation (SD). Criterion of significances was set as p<0.05. Kolmogrov-Smirnov test was used to describe normal distribution. Pearson’s correlation coefficient test was used to determine intra-rater reliability of variables.

## 3. Results

The demographic features of subjects were listed in Table 1.

The result of kolmogrov-smirnov test showed that all of variables include MF muscles thickness, CSAs, bladder diameter at rest and during PFM contraction had normal distribution (p> 0.05).

Pearson’s correlation coefficient test was used to determine intra-rater reliability of one investigator for MF muscles thickness, CSAs, bladder diameter at rest and during PFM contraction (Table 2 and Table 3). Average measure of ICCs values with 95% CI and the standard error of the measurement (SEM) were calculated within investigator (Table 2 and 3). These ICCs indicate good to excellent agreement for a single investigator between two times measurement. The results showed that the intra-rater reliability of bladder wall displacement was high (ICCs for rest and PFM contraction state: 0.96 and 0.95 respectively). The intra-rater reliability of the MF muscles CSAs at the L4 level was good to high (ICCs 0.75 and 0.91 for right (Rt) and left (Lt) side respectively). For the rest thickness of MF muscles at the L5-S1 level the intra-rater reliability was moderate to high (ICCs: 0.84 and 0.67 for Rt and Lt side respectively) and moderate for the L3-4 level (ICCs: 0.68 and 0.66 for right and left side respectively). But, the intra-rater reliability of the MF muscles contraction thickness during task1 was high for the L5-S1 level (ICCs: 0.85 and 0.87 for Rt and Lt side respectively) and good to high for the L3-4 level (ICCs: 0.81 and 0.74 for Rt and Lt side respectively). Also, the intra-rater reliability of the MF muscles contraction thickness during task2 was high for the L5-S1 level (ICCs: 0.84 for both sides) but moderate to good for the L3-4 level (ICCs: 0.64 and 0.72 for Rt and Lt side respectively).

**Table 2:** Intra- Rater Reliability of the variables

		Repeat 1	Repeat 2	Intra-class Correlation Coefficient	95% Confidence Interval		SEM^b^	SEM^c^

					Lower Bound	Upper Bound		
**Bladder Diameter: rest, pelvic floor muscle contraction (mm)**	Rest	40.20±15.76^a^	38.03±13.65	0.961	-0.623	4.961	4.37	3.78
	Cont	35.52±16.82	34.65±13.97	0.958	-2.327	4.065	4.66	3.87
**Multifidus Cross Section Area, L4 level (cm^2^)**	Rt	85.20±20.19	88.23±22.59	0.744	-12.367	6.321	5.60	6.26
	Lt	78.99±22.59	82.89±19.28	0.911	-9.590	1.790	6.26	5.35
**Multifidus Rest thickness, L5-S1 level(mm)**	Rt	29.87±4.79	30.68±5.64	0.84	-1.76	0.134	0.74	0.87
	Lt	29.23±6.80	31.20±6.33	0.67	-3.64	-0.297	1.05	0.98
**Multifidus Rest thickness, L3-L4 level(mm)**	Rt	29.25±4.25	30.47±4.21	0.68	-2.26	-0.166	0.65	0.65
	Lt	29.82±4.09	30.58±4.18	0.66	-1.82	0.303	0.63	0.65

a Values are Means and Standard Deviation.

b Standard Error of Measurement, Repeat 1

c Standard Error of Measurement, Repeat 2

**Table 3:** Intra- Rater Reliability of the variables

		Repeat 1	Repeat 2	Intra-class Correlation Coefficient	95% Confidence Interval		SEM^c^	SEM^d^

					Lower Bound	Upper Bound		
**Multifidus thickness L5-S1 level during Task1^a^(mm)**	Rt	33.92±5.56^b^	34.64±5.94	0.85	-1.53	0.459	0.86	0.92
	Lt	34.75±6.01	35.73±6.08	0.87	-1.94	-0.026	0.93	0.94
**Multifidus thickness L3-L4 level during Task1(mm)**	Rt	33.61±4.47	34.74±4.34	0.81	-1.96	-0.285	0.69	0.67
	Lt	35.21±4.16	35.84±4.44	0.74	-1.60	0.352	0.64	0.68
**Multifidus thickness L5-S1 level during Task2 ^a^(mm)**	Rt	34.82±5.65	35.69±5.29	0.84	-1.84	0.109	0.87	0.82
	Lt	35.81±5.32	36.80±6.24	0.84	-2.05	0.064	0.82	0.96
**Multifidus thickness L3-L4 level during Task2(mm)**	Rt	35.37±4.30	36.32±4.42	0.64	-2.12	0.204	0.66	0.68
	Lt	35.78±4.13	37.02±4.69	0.72	-2.291	-0.189	0.64	0.72

a Task 1= Arm elevation with glenohumeral joint at 125 degree of abduction and elbow at right angle, without weight at hand, Task 2= Arm elevation with glenohumeral joint at 125 degree of abduction and elbow at right angle, with weight (0.5 kg) at hand,

b Values are Means and Standard Deviation,

c Standard Error of Measurement, Repeat 1,

d Standard Error of Measurement, Repeat 2.

## 4. Discussion

Based on the findings of this study, there were moderate to high intra-rater reliability of RUSI for measuring the MF muscles CSAs, rest and contraction thickness, and bladder wall displacement as a reflection of PFM action (ICCs: 0.64- 0.96).

### 4.1 Reliability of the TA method of RUSI

The results showed that the intra-rater reliability of RUSI for bladder wall displacement was excellent at rest and during PFM contraction. (ICCs for rest and PFM contraction state: 0.96 and 0.95 respectively). The ICC values indicate that the between two times measurement intra-rater reliability was excellent. The SEM indicated that there was little variability due to the operator as the plane of measurement. Our findings for intra-rater reliability of bladder displacement was same as Sherburn et al. (2005). The ICCs of present study were better than Sherburn et al. (2005) (0.96 and 0.95) (Sherburn et al., 2005). Also, in contrast of Sherburn et al. (2005) the SEM of the present study had little variability. Also, the ICCs values of the present study was very similar to Thompson et al. (2005) for PFM contraction (0.93). In contrast of our study, the SEM of Thompson’s study was low (0.13) and they were recorded images at sagittal view. However, in the present study, the transverse view was used to record images of the bladder wall displacement. Therefore, we conclude that TA method of RUSI is a reliable method to quantify the PFM contraction in healthy subjects.

### 4.2 Reliability of RUSI for Measuring the MF Muscles CSAs

Based on the results of the present study, the intra-rater reliability of the MF muscles CSAs at the L4 level was good to high (ICCs 0.75 and 0.91 for right (Rt) and left (Lt) side respectively). Also, the amounts of SEM were low and within normal range for MF muscle CSAs (SEM= 5.35 to 6.26). This finding was same as the study of Stokes et al. (2005) (The ICCs for multifidus CSA ranged between 0.98 and 1.00) (Stokes et al., 2005). Their study design was different from our study aspects of demographic characteristics of participants, side of measurement (they measured only left side), and time between the two repetitions (one week apart in a blinded fashion, at the same time of day by one operator). (Stokes et al., 2005). Also, our finding was in accordance with the finding of Pressler et al. (2006) (the between-day intra-rater reliability for the RT and Lt S1 multifidus muscle was ICCs = 0.80 and 0.72 respectively). However, the ICCs for the present study were higher than the study of Pressler et al. (2006) and we used L4 level of the MF muscles for intra-rater reliability. Indeed, the participants in the study of Pressler et al. were female. In the present study, reliability of the RUSI for measuring the MF muscles CSAs accomplished at two sides of lumbar vertebrae. Also, two genders participated in this study. So, it can be concluded that RUSI for measuring the MF muscles CSAs in healthy subjects is a reliable method.

### 4.3 Reliability of RUSI for Measuring of the MF Muscles Thickness

For the rest thickness of MF muscles at the L5-S1 level the intra-rater reliability of the present study was high to moderate (ICCs: 0.84 and 0.67 for Rt and Lt side respectively) and moderate for the L3-4 level (ICCs: 0.68 and 0.66 for right and left side respectively). But, the intra-rater reliability of the MF muscles contraction thickness during task1 was high for the L5-S1 level (ICCs: 0.85 and 0.87 for Rt and Lt side respectively) and good to high for the L3-4 level (ICCs: 0.81 and 0.74 for Rt and Lt side respectively). Also, the intra-rater reliability of the MF muscles contraction thickness during task2 was high for the L5-S1 level (ICCs: 0.84 for both sides) but, moderate to good for the L3-4 level (ICCs: 0.64 and 0.72 for Rt and Lt side respectively). The amounts of SEM were low and within normal range for all of outcome measure of MF muscles thickness measurements (SEM= 0.64 to 0.94). So, the errors of measurement for MF muscles thickness were low and our measurements were at standard levels.

The results of the study for task 2 were consistent with Koppenhaver et al. findings (Koppenhaver et al., 2009). They evaluated the intra-examiner reliability of RUSI in obtaining thickness measurements of L4 level of the MF muscles at rest and during contractions (task 2) in patients with LBP and they found intra-examiner reliability ICCs ranged from 0.87 to 0.98 for between-day comparisons. However, Participants in Koppenhaver et al. (2009) were non-specific CLBP and L4/L5 level was used for MF muscle thickness measurement. In the present study, participants were healthy subjects and two levels (L3/L4 and L5/S1) were used for evaluation of MF muscles thickness. Also, the results of the present study for intra-rater reliability of the MF muscle thickness at rest state were consistent with findings of Wallwork et al. (2007) (ICCs = 0.95; 95% CI: 0.86 to 0.99). In contrast of this study, they investigated intra-rater reliability of lumbar MF muscle thickness at L2-3 and L4-5 level.

In present study, the ICCs for the L3-4 level of vertebrae were smaller than L5-S1 level. Also, the ICCs for L3-4 during task1 were greater than the ICCs for task 2. The possible reason that proposed for these findings is that we measured two levels of the lumbar vertebrae at same image and the mid-point of image was at the L4-5 level, so the curvature of the image may be resulted to differences in the amounts of ICCs for two levels of lumbar vertebrae. Also, the person who was in charge of RUSI measurements was physiotherapist with only 3 month experiences. So, it may be possible to collect more reliable data with more experiences in this field. However, based on the finding one can conclude that RUSI is a reliable method to measure the MF muscles thickness at rest and during functional tasks in healthy subjects.

## 5. Conclusion

Based on the findings of this study, there were moderate to high intra-rater reliability to measure the MF muscles CSAs, the MF muscles thickness at rest and during contraction, and bladder wall displacement as a reflection of PFM action through RUSI. Therefore, we conclude that TA method of RUSI is a reliable method to quantify the PFM contraction in healthy subjects. Also, RUSI is a reliable method to measure the MF muscles CSAs, the MF muscles thickness at rest and during functional tasks in healthy subjects.
